# Possibilities for maintaining a strong self – a grounded theory study of relational experiences among Thai women in Sweden

**DOI:** 10.1080/16549716.2017.1396881

**Published:** 2017-11-09

**Authors:** Cecilia Fernbrant, Anette Agardh, Maria Emmelin

**Affiliations:** ^a^ Social Medicine and Global Health, Department of Clinical Sciences, Lund University, Malmö, Sweden

**Keywords:** Thai women, international marriages, migration, controlling relationships, social support

## Abstract

**Background**: Due to increasing globalization and Internet communication, the number of international marriages has increased. In Sweden, 75% of the Thai population are women, among whom 80% are partnered with Swedish or other Scandinavian men. Previous studies have indicated that lack of autonomy, social isolation, and stigma are important risk factors for poor mental health for foreign-born women as well as for women in international marriages.

**Objectives**: To explore what characterizes the processes, choices, challenges and relational conditions that Thai women, partnered with Swedish or Danish men, experience during their first years in Sweden.

**Method**: A qualitative study using a Constructivist Grounded Theory approach based on fourteen individual interviews with Thai women partnered with Swedish or Danish men and residing in Sweden.

**Results**: The core category ‘possibilities to maintain a strong self in Sweden’ is linked to five categories characterizing the process that the women go through over time. The subcategories illustrate different paths taken even if there were possibilities to change paths along the way. The women had, for different reasons, reached a turning point that made them leave Thailand. In Sweden, they started in dependency and struggled in different ways to adjust to relational norms and handle prejudice. Toward the end of the timeline, differing ways of recognizing life choices depended on access to social networks and partners’ attitudes.

**Conclusion**: Our study showed the crucial role of economical, emotional and social support from partners and networks for Thai women’s possibilities to maintain a strong self and good health after migration. This implies a need for supporting Thai women to be more independent by providing access to language education, employment and community involvement. The current requirement for becoming a permanent resident should also be reviewed not to jeopardize women international marriages possibilities’ to leave unhealthy relationships.

## Background

Increasing globalization and Internet communication have led to a global increase in international marriages. International marriage is a term present in legislated acts and used to designate a marital relationship between two parties of different nationalities, where only one of the parties resettles [1]. Persons who migrate as spouses are primarily women, and the main countries they come from are China, Vietnam, the Philippines and Thailand [2,3]. The main receivers of foreign spouses in the world are Taiwan, Singapore and South Korea [3]. Unfortunately, there has also been an increased commercialization of arranged international marriages, which in turn increases the risk of trafficking and commodification [4].

International marriages are also prevalent in Sweden, a country with the largest number of immigrants per capita in Europe [5]. According to Swedish statistics, Swedish citizens who enter into international marriages often marry Thai or Iraqi women, followed by Somali, Serbian and Turkish [6]. In Sweden, 75% of the Thai population are women, and among these, 80% are partnered with Swedish or other Scandinavian men [6]. According to Swedish legislation, Thai women, or other third-country nationals, who marry a Swedish resident receive a two-year temporary permit of residence, after which a permanent residence may be given if they are still partners [7]. During this period the woman’s main source of economic support is her partner’s income, i.e. the Swedish resident, in contrast to, for example, refugees who can get support from social services upon arrival. In addition, it is difficult for newly arrived Thai women, who do not speak Swedish, to get a job.

International marriages are exposed to prejudice or discrimination to a larger extent than other couples, especially spouses from non-Western European or North American countries, including Thai women [5]. Prejudice or stereotype ‘is a conscious or unconscious held belief or expectation about a group of people that does not easily permit exceptions and usually helps a group that produces stereotypes to feel better about itself’ [8]. The most common prejudice about Asian women involved in international relationships is, for example that they are exotic, submissive, sexually available, domestic, mail order brides, sexual slaves or prostitutes [4,9–11]. There is less prejudice against men than against women; however, men in international marriages are to some extent seen as unsuccessful males who lack the ability to find a partner in their home country [12].

Prejudice may lead to adverse consequences such as discrimination and social exclusion, risk factors for social isolation and poor mental health. Previous research shows that social isolation is strongly related to poor mental health [13]. Foreign-born women (vs. native-born women) have increased risks for poorer mental health, including severe mental illness [14]. Moreover, in a Dutch study, lack of autonomy and personal freedom were observed as risk factors for increased suicidal behavior among foreign-born women, including South Asians [15]. For new members of a society, employment and social participation are important ways to reduce the risk for marginalization and/or social exclusion. A study from Australia showed that Thai women married to Anglo-Australian men reported social isolation as a main concern [16]. Thus, Thai women residing abroad may be at risk for social isolation, due to poor language skills, lack of contact with other close relatives and/or other persons in the surrounding society, factors that also make Thai women exposed to intimate partner violence (IPV) especially vulnerable [13,15,17,18].

Since lack of autonomy, social isolation and stigma have been shown to be risk factors for poor mental health among foreign-born women as well as women in international marriages [18], these concepts formed the basis for the theoretical framework used in this study. The role of autonomy as a basic need has been discussed by several philosophers [19–21]. Doyal and Gough believe that there are universal basic needs for human development, rights and well-being. Their theory builds on a listing in hierarchical order, starting with universal goals (normative and ethical reasoning), through basic needs (health and autonomy), and ending with intermediate needs (subjective basic needs differing between cultures) [19,22]. On the other hand, according to Sen, there are no universal basic needs pertaining to all people; instead, basic needs are dependent upon cultural and situational aspects [20]. In contrast to Sen, Nussbaum has, in overall agreement with Doyal–Gough, provided a comprehensive list of central human functional capabilities relating to autonomy where bodily integrity, practical reason (planning one’s own life), and affiliation (belonging to a context) are the most significant [21]. According to Gough [22], the Doyal–Gough theory can be used to balance Nussbaum’s more extended approach to capabilities and well-being with Sen’s sparser version. Social isolation can be seen in relation to the theoretical concept of social capital. According to Putnam, social capital is a characteristic of communities based on social participation, trust and reciprocity norms, while others have emphasized its role as an individual resource based on individuals’ involvement in social networks [23,24]. On both community and individual levels, social capital has been shown to have a positive impact on health [18,25]. Lack of social participation may result in social isolation. However, it may then be important to distinguish between participation in different types of networks: bonding (social groups similar to one self), bridging (social groups differing in terms of social identity), and linking (social groups with access to power and authority), as suggested by Szreter and Woolcock [26]. Stigma refers to being treated differently based on attributes, traits or behavior [27]. Goffman distinguished between three different types of stigma, i.e. relating to body, character or social collectives. Goffman further discusses how passing and covering can be used to handle exposure to stigma [28]. The strategy of passing includes controlling information by trying to blend into the ‘normal’ group as much as possible, and covering is a strategy where a person with a potential stigma attribute tries to avoid disclosing it [27]. Thus, the potential interplay between these various concepts provided the rationale for the current qualitative study of Thai women living in international marriages in Sweden.

Research on international marriages between Swedish men and Thai women is scarce, and little is known about the health, social support and exposure to IPV among Thai women residing in Sweden. However, our previous study, based on a public health questionnaire, examined the prevalence of IPV, social isolation and social capital among Thai women partnered with Swedish men in Sweden and the association with mental health. The results showed that Thai women had high levels of social isolation, low general trust and that they had experienced partner violence but more often in their country of birth than in Sweden [18]. To further understand the underlying mechanisms and processes that could explain the observed associations, a Grounded Theory approach was used, since it is particularly useful for generating explanatory models concerning complex social phenomena [29]. Based on our theoretical framework, stigma, autonomy and social capital were used as sensitizing concepts, implying that they did not steer us in what to see; instead they suggested directions where to look [30,31].

This study aims to explore what characterizes the processes, choices, challenges and relational conditions that Thai women partnered with Swedish or Danish men experience during their first years in Sweden.

## Method

### Study design

The study design was qualitative, using a Constructivist Grounded Theory approach [29] aimed at producing a theoretical understanding of processes, choices, challenges and relational conditions involved in the studied phenomenon. Individual in-depth interviews were chosen for data collection, since they are suitable for exploring and understanding participants’ own experiences and appropriate for the sensitive nature of this study [32].

### Study setting

This study was performed in Skane county, situated in southern Sweden. Participants were recruited from Malmö, Lund, and Helsingborg, the three largest cities in Skane county, in southern Sweden. Malmö is the third largest city in Sweden (280,000 inhabitants) and the largest city in Skane. Helsingborg is the second largest city (139,000 inhabitants), and Lund (117,000 inhabitants) is the third largest city in Skane county. Sweden has some 39,000 Thai residents, of whom 31,000 are women. Approximately 1,000 Thai women reside in Skane county, and among those, 95% are partnered with Swedish or Danish men [6].

### Study population and selection of informants

The informants were initially purposively sampled among Thai women who previously had taken part in a public health survey and who had indicated their willingness to participate in an interview study, by supplying their names and phone numbers [18]. The purpose was to reach women in different age groups and with different socio-economic or educational backgrounds who were residents in Sweden and partnered with Swedish/Danish men. We also wanted to include women both with and without relational experience of controlling or violent behavior. This recruitment was somewhat hampered by the fact that data collection started 2 years after the survey had ended. This meant that phone numbers could be inactive or that women no longer wanted to participate in an interview. However, this sampling procedure later facilitated a snowball sampling [32], whereby the initial informants could guide us to other women with characteristics or experiences that could help us to theoretically saturate the emerging analysis. For practical reasons, we limited the sampling of informants to those living in the three largest cities in Skane count.

In all, 14 individual in-depth interviews with Thai women were conducted. The women had been in Sweden for an average of 5 years, and their age ranged from 29 to 63 years. Educational background varied, with seven of the women having finished high school, five having attended university and the rest having seven or less years of schooling. Twelve of the women had jobs in Sweden, all within the service sector. Of the remaining two, one was studying and the other was unemployed. Seven of the informants had met their husbands in Thailand, one on the Internet, while the others had met their partners after coming to Sweden. Six of the women had left their first partner in Sweden and were currently living with a new man. Six of the women had left children behind in Thailand and six women had given birth in Sweden.

### Data collection

The first author (CF) performed all interviews, the main part from May to November 2014 and two additional interviews in August 2016. Informants were first contacted via phone and informed about the objectives of the study. If they accepted to take part in an interview, they had the opportunity to choose the time and place for the meeting. Most informants accepted the invitation to come to the interviewer’s workplace where an office was arranged for interview purposes. Only one of the informants wanted to meet in another location. Prior to the actual interview, informants received an information letter and signed a consent form. The interview guide was arranged under four broad themes, i.e. health and well-being, family life, social relations and support (from other family, friends and social networks). The interview guide was flexible, and through open-ended questions and probing, efforts were made to capture the women’s life journey focusing mainly on the time after they had decided to leave Thailand for a new country. The interviews ranged in length from 45 minutes to 2 hours and were audio-recorded. The interviews were conducted in Swedish, English or Danish.

### Data analysis

**Figure 1.**
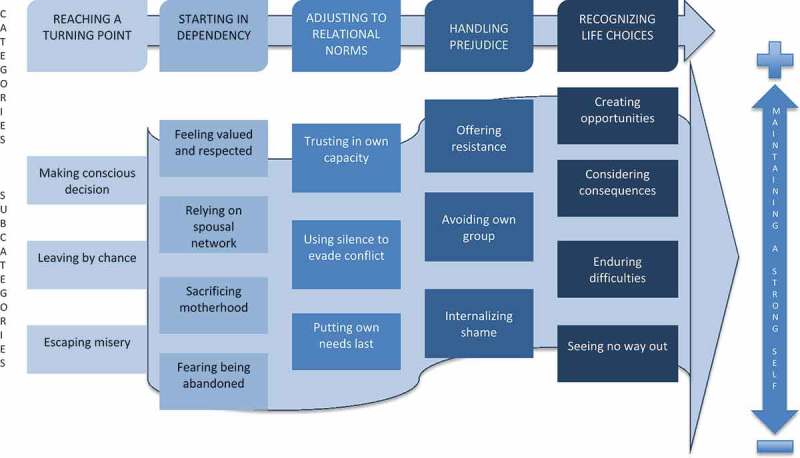
Theoretical model of different possible pathways for Thai women partnered with Swedish/Danish men in Sweden.

During the data-collection period, the interviews were continuously transcribed and subjected to preliminary coding. Coding was carried out according to Charmaz’ Constructivist Grounded Theory [29] with the stages of initial, focused and theoretical coding. Memos were written in connection with each interview as well as during the coding process. Memos helped to describe emerging categories that were continuously discussed in the research group. Box 1 gives an example of a memo written in the beginning of developing one of the categories, ‘Starting in dependency’. The analysis involved a constant comparison and oscillation between data and evolving categories. The analysis resulted in the construction of a core category with its supporting categories and subcategories describing properties and dimensions. The analysis led to the development of a theoretical model containing different possible pathways, based on interpretation of informants’ experiences (Figure 1).

## Results

### Maintaining a strong self

The analysis came to be focused around the core category ‘Maintaining a strong self’, which characterized an aspiration as well as a possible outcome of the social process of migrating to Sweden by means of entering into a relationship with a Swedish/Scandinavian man. However, the possibility of maintaining a strong self was highly dependent on previous circumstances, formal requirements, own and others’ expectations, and available supporting networks. Figure 1 gives an overview of the developed theoretical model. The five main categories, *Reaching a turning point, Starting in dependency*, *Adjusting to relational norms*, *Handling prejudice* and *Recognizing life choices*, illustrate steps in the overall process of encountering a new setting. The subcategories then illustrate key aspects of alternative pathways that may lead to higher or lower chances, of maintaining a strong self. These should be seen as different ‘ideal paths’, and not as descriptions of individual women. As seen in Figure 1, these paths are not fixed and it is possible to change paths along the way. However ending in one of the lower paths is seen as giving less chances of maintaining a strong self. The results detailed below are presented under the headings of the main categories describing, through subcategories (in bold), their properties. Quotes are intertwined in the text to support how the interpretation is grounded in the data.

### Reaching a turning point

Before deciding to leave Thailand for Sweden the women had all reached a turning point where they felt they had to make a choice. Leaving family, friends, work and possibly children behind was a great decision not made easily. Reasons for leaving ranged from escaping misery from a dangerous situation to making a conscious decision together with the partner.

Leaving Thailand to live in a new country with a man was, for women ‘making a conscious decision’, based on extensive information and good knowledge of what Sweden had to offer as well as a solid relationship with a long-term boyfriend: ‘We wanted to get to know each other and I had a good job in my home country, so why the rush to move to Sweden’ (F12).

Another possible way for women to move to Sweden was by invitation, either by a man with residence in Sweden or by another contact living in Sweden. The women ‘leaving by chance’ had not known their partners for long, and one woman said: ‘you should take the chance if somebody is friendly/nice’ (F11). In this case, the women did not have much knowledge of Sweden or what to expect and made their decisions to leave based on a man’s invitation, their perceived responsibility for their parents and children or other practical reasons. Even though leaving Thailand was optional, the women were hoping for greater opportunities and a better life in Sweden. ‘I wanted to get away from the Thai society. I wanted to improve my life. I did not just come for the money’ (F8).

Partner abuse was a strong reason for wanting to escape a current or former partner and starting a new life somewhere else. Being forced to leave the country often meant that there was no safety net on the other side but that it was the only option to ‘escape misery’ in an untenable situation. Getting away from an abusive relationship was one reason for leaving Thailand. ‘When I got here I had no intention of meeting a Swedish man, I just wanted to get away’ (F1).

### Starting in dependency

All informants started their life in Sweden in dependency due to the fact that in order to be allowed to stay, they needed to have a Swedish citizen willing to support them financially for 2 years. To be able to work in Sweden, the women needed to have a job and permission to work before they arrived. These terms made it difficult for them to provide for themselves upon arrival and left them depending on their partner. Such dependency was difficult to break and easily created long-term dependency. Due to the type of relationship the women were in, they experienced the situation differently.

Living in dependency was not necessarily a negative experience if the women were ‘feeling valued and respected’ by the partner. A woman who had waited 7 years before she moved to her boyfriend in Sweden argued that: ‘he doesn’t oppress me and I don’t oppress him, we respect each other, that’s what matters. Many people don’t have mutual respect. But when it comes to money, I’m still dependent on his money’ (F12). The analysis showed that the greatest likelihood for mutual respect was in relationships where the woman knew her partner before moving in with him, and had made an active choice of doing so:

My husband is different. He wants me to go to school and be part of the society. He doesn’t want a woman like the others [Swedish men married to Thai women] do. The others just want Thai women that they can have sex with whenever they want and have a clean home. They are slaves, cook, wash clothes, anything, but not my husband. I don’t have to cook if I’m not eating. (F1)

Entering a new social context in a foreign country without being able to speak the language was difficult. A good way of becoming socially accepted in the new society was to be accompanied by some native person, and thus the women often had to ‘rely on spousal network’ for social interactions. However, the informants complained that their partners’ social contacts often were few and hence their social participation as a couple was low. The women were either alone, relying on their partners’ company, or seeing other Thai women. The women felt lonely and isolated but accepted the situation, sometimes as a mutual agreement. ‘You do everything to make the men happy, men who can take care of you. It’s a beneficial situation for both. If you give me a better life, I will take care of you’ (F8). But social isolation could also be explained by lack of time for social activities: ‘No, I don’t have much time. I work and then I take care of the children. And I don’t even have energy to study. I’m so tired’ (F4).

It was apparent that the possibility to start all over in a new country came with a high price. Some of the women had had to leave previous children behind in order to seize an opportunity when it came up. Both due to financial aspects and due to the wishes of the new partner, women ‘sacrificed motherhood’ by leaving their children with relatives in Thailand. They were hoping to go back for them later on; however, it might take several years and there was no guarantee that they would ever succeed. One woman described her situation when she finally had saved enough money to bring her child to Sweden: ‘I had given up custody in order for my son to stay with them [parents of the father of the child] and when I came to pick him up, the grandmother wouldn’t let him go’ (F8). The woman’s former parents-in-law had got accustomed to the money they had received for taking care of her son and would not let her have him back. Thus, the woman had to accept a life without her children. Another woman who had lost contact with her child after moving to Sweden, but still was hoping to bring him, stated: ‘It’s hard because you know, he’s only two and when he starts school everybody will have a mom to pick them up, you know, but he won’t’ (F9). The results indicate that some informants had had to leave their children behind, in their efforts to, for example, provide financial support for their children and parents or to escape a violent relationship. However, to compensate for children left behind, they wanted new children with the Swedish man. This wish was sometimes not mutual in the relationship: ‘I want a child but my husband says maybe maybe, and that he has to get a good job first’ (F13).

The two-year rule resulted in ‘fear of being abandoned’ and thereby not being able to stay in Sweden. This fear led to submission and could also result in difficulties to communicate with the spouse. ‘I want us to be a family, all to have the same rights, but still I don’t know what I’m supposed to do. I can’t breath and if I do it’s wrong’ (F8). Women who had left Thailand in order to escape misery had a stressful situation and were often sent to live with a relative in Sweden. They were then dependent upon their relatives for financial support and a two-year residency. One woman had just arrived and was living with, and working for her aunt in Sweden. Without consulting or informing her, the aunt tried to sell her to a Swedish man, thus illustrating one of the more adverse consequences of being dependent and lacking other social relations:

She [the aunt] wanted him to marry me, she wanted 25,000 USD from him. First I went to see him and she left me alone with him in his home. He wanted to have sex with me, I said no I don’t want to. I said we are not getting married, even though you have a lot of money, I don’t want that. (F4)

The woman refused to marry the man and ended up in a serious conflict with her aunt who even contacted her former abuser in Thailand and disclosed her hideout. Fortunately, shortly thereafter, she met another Swedish man whom she liked and who was willing to take her in.

### Adjusting to relational norms

All informants were motivated and excited to build a new life in Sweden. However they had also realized that moving to a different culture and being dependent upon relatives or a man more or less unknown to them required flexibility. The analysis identified three main paths/categories, one where the women were strengthened after migration and trusted in their own capacity, another where they avoided arguments and speaking their minds, thus evading conflict by silence, and a third where they were docile, serving their husband and putting their own needs last.

The participants who were ‘trusting in their own capacity’ experienced that women had higher status in Sweden than in Thailand. After moving to Sweden they had understood why:

Swedish men fall in love with Thai women because we are humble, different attitudes than Swedish women. Before I came to Sweden I had heard that Swedish women were strong and cold. Then when I came here I realized that it’s because they have the same rights as men. (F8)

They also felt that questioning decisions was legitimized in contrast to in Thailand where one did as one was told. The women were raised in a society where children and young people were not allowed to question information and orders.

I like Swedish culture and I’ve learned a lot from your culture. I’ve learned that it’s ok to ask why, when you learn new things in Komvux [school for grown ups]. I’ve also learned to explain why things are like they are. It’s not like in Thailand where you have to respect and believe your parents without learning why. You can’t love anyone or respect them without a reason. (F2)

Due to this new context, they felt stronger and more confident than before they had moved to Sweden. They had started to speak their minds and to decide for themselves what was in their favor. Some of the women even developed the strength to confront men with controlling behavior. ‘We discuss and talk to each other about that I don’t like when he does that. He doesn’t have to call all the time and ask me where I am, what I’ve done and what I’m going to do. Today, tomorrow, later on’ (F6).

However, ‘conflict could also be evaded by silence’. In Thailand retired parents economical needs are the responsibility of grown up children due to lack of a national social security system. Thus, the informants were expected to send money home in order to support their parents. Such normative divergence between the Thai and the Swedish systems could cause conflict within a relationship. It was difficult, especially in the beginning before the women were earning their own money, for them to help their parents without their husbands’ consent.

I help my mother if she needs money. I send to her sometimes. He doesn’t understand. He thinks she should be able to take care of herself. But we don’t have money for retired people in Thailand, the children are expected to support their parents. (F10)

In the interviews it was prominent that such conflicts were evaded by not talking about it, or that the women could not start to send money until they got a job of their own.

I give 250-300 USD to my father every month, but I can’t tell my husband. He won’t understand, he can’t accept why you should give money to your parents, it’s the parents that should give to the children instead. We’ve talked about this but he won’t understand. (F12)

Adjusting to fit could also mean ‘putting their own needs last’. Some of the women described themselves mainly as caretakers of husbands, children and home. ‘Swedish men want Thai women because they don’t talk back, they just accept and do what the men want – take care of the home, clean and help them’ (F1). The women were struggling to satisfy everybody and to prove that they were good wives by putting everyone else’s needs before their own. The home was their domain, and they explained that their qualities of being a good housewife made them attractive to Swedish men, even though they occasionally would like to have some more help from their men.

Well, sometimes I would like, you know, him to help out a bit. But I don’t know, I think it’s normal. Sometimes we have some problems and sometimes we don’t. I let him free. I don’t want to nag and tell him when to come home or make a list of what he has to do when he comes home, no. (F9)

## Handling prejudice

The women were met by suspicion and condescending attitudes in Sweden due to prejudice against international marriages/relationships between Thai women and Swedish/Western men. The rumors behind such attitudes were according to the informants based on views that all Thai women in such relationships were prostitutes or adventuresses.

I think when they see you are Thai they just think you’re easy or maybe they think you will be like those Thai girls in the bar they meet, or maybe they heard so much bad about Thai women so they just think you are the same, I don’t know. (F9)

All informants had experienced glances and frowns, and sexual harassment was also common. According to the informants, prejudice and discrimination expressed as sexual harassments or physical assault decreased with time, which may have been a result of different coping strategies developed.

One way of dealing with prejudice was not to accept it and instead ‘offer resistance’ to such attitudes. This required strength and confidence as well as support and understanding from others. One of the participants was spat on by a woman in the train and was so surprised and taken off guard that she was not capable of saying anything. This time she was passive and later regretted it: ‘I didn’t have the energy to react and ask her why did you do that? Tell me, talk to me, why? […] If I see her again I will go and ask her, what do you want?’ (F3). But she also recalled another occasion when she was more prepared and just by the look in her eyes managed to chase away a man who intimidated her, when: ‘He cranked down his window [of the car] and said Hi. I said Hi, and he asked ‘Do you want to suck my dick for five dollars?’ I remember those words’ (F3). This woman had the support of her husband and was part of the local Thai community. She was trusting in her own agency and had found a stronger self after moving to Sweden.

Another strategy used was to ‘avoid other Thai women’. This meant pretending not to be a part of the Thai community and only engage with Swedish or other foreign-born persons. The informants described a high level of distrust and talking behind each other’s backs within the groups of Thai women. The participants did not identify with the rumors and prejudice for their own sake; however, they believed it to be true for other Thai women in Sweden. ‘In my opinion Thai women don’t help each other, they suppress each other. They want to exploit one another, they do’ (F1). The experiences from the informants all confirmed that Thai women could be mean to, and compete with each other, and did not always support one another:

Thai women talk behind each others’ backs and show off their new things and their men. Look what I have! If you have a good man they try to take him. My first husband cheated with my friend. (F14)

A third way of handling prejudice was to accept and ‘internalize shame’ by accepting other people’s opinions of Thai women.

I can’t stop their thoughts [the Swedish people with prejudice] and you need to understand that it’s like that. I can’t change them, but it’s true, many [Thai women] are like that. When you go to Harry’s [a bar in Sweden] you only see Thai women there trying to talk to Swedish men. (F12)

The strategy of internalizing shame resulted in women looking down upon themselves, losing self-confidence and degrading themselves.

## Recognizing life choices

When moving to Sweden, the informants were more or less aware of the risk of expecting and hoping for something that might not turn out the way they wanted. It was first after having resided in Sweden for a while that they ‘recognized what different life choices’ their new situation had to offer. Depending on the women’s situation with regard to the relationship to partner, Swedish language skills, job situation and social support, the women’s possibility to choose varied.

At best the women felt that they were ‘creating opportunities’ and remained strong, as illustrated at the end of the first path. The challenges in the relationship were manageable and they wanted to stay with their partner.

Yes, I want to be with him, because I know he’s the best. He’s mine, not perfect, but… he’s the person I want to live my life with, that I want to have children with, I’m rather sure of that. You know when you fight many times you try to say this was the last time I’m going to let him do that to me. And then you think what if something happened to him, I couldn’t take that neither physically or mentally. That’s what it’s all about when you love someone. […] Just seeing him close by makes my heart warm, that’s he is my boyfriend. (F12)

This path thus demanded support from husbands and others to learn Swedish or encouragement to continue education to get a good job. Getting this support created an opportunity for the women to decide for themselves with regard to different life choices.

In the next path, women would be prepared to consider consequences present in their relationship and make a conscious decision whether to stay or not.

But he’s not that kind, you know. I see some Swedish guys with prams, you know taking care of the kids, going the park but he’s not [like] that. I have to tell him what to do, like when we invite a family for dinner. I’ve been cooking the whole day and when we finish dinner I have to say like okay your turn, you have to get the plates and so on and you know I have to tell him every detail of what he has to do. (F9)

This illustrates a situation where the relationship was acceptable but the women still had other social contacts that made it possible to feel like the move had given increased opportunities for maintaining a strong self. Other experiences indicated the possibility for women to actually leave abuse relationships and thereby retain their self-respect.

We fought and he just came up and hit me. I tried to push him away, but he continued to hit and kick and also hit my son […] When he calmed down I called the police immediately and my friend came and picked me and my children up. They had to find a place for us to stay. (F10)

The end of the third path illustrates a situation where the women felt limited possibility to choose a better option. The women stayed with their partners, sometimes much older, even though they would have preferred to leave and ‘endured difficulties’.

It is very difficult to change [him]. Very difficult to start over and it’s so boring to be with him. We have difficulties understanding each other. […] I just want to leave. I much rather live by my self. (F5)

In this situation, the women were free to leave but lacked resources to do so. They may also have felt sorry about leaving an older spouse alone and stayed because of pity, putting their own needs last. ‘Instead of being alone he rather have me there. I clean, I wash and I iron clothes for him, I do everything for him. So he doesn’t have to’ (F5).

The last path theoretically ended in a situation characterized by lack of opportunity to choose. Controlling or violent partners, combined with lack of opportunity to provide for themselves, were the main factors involved. Poor language skills, lack of social contacts, mainly having taken care of the home, could be contributing to this situation making the women feel isolated and homesick and having low level of trust in other people. They were left in long-term dependency ‘seeing no way out’. Among our informants, there were few women representing this path. However, there were experiences that clearly indicated this path as a possible scenario for others.

He hit her in front of her child. It was terrible when he hit her, I felt so bad. I couldn’t do more. I wanted to smash the door and get her out of there. But she’s an adult. She knows the language, she’s very good. She’s just used to violence and that’s why she’s staying. (F3)

## Discussion

The Grounded Theory analysis made possible a theoretical understanding of the underlying mechanisms and processes involved in the associations between mental health and social circumstances. The core category, to maintain a strong self, was seen as a common challenge among the women and the outcome depended upon different social processes described as alternative pathways after migration. The women had all reached a turning point that made them leave Thailand. In Sweden they started in dependency with a partner more or less known to them, and they were trying to adjust to fit in. The pathways they then took were interchangeable and they had the possibility to change lanes along the way. They had different ways of handling prejudice from the Swedish society, and their life choices depended very much on their access to social support and their partners’ attitudes. Our findings are in line with previous studies, yet the specific focus on Thai women in Sweden adds to the existing body of research within the field of international marriages [1,3]. Additional contributions of the study are the identification of the different coping strategies used to handle stigmatization and the notion that lack of autonomy may result in negative health consequences, through social isolation and lack of social capital.

The philosophical discussion concerning human capacity and basic needs described in the theoretical framework can be applied to the health and well-being of Thai women in Sweden [19,20,21,22]. Our results show that in order to develop and be integrated into the Swedish society Thai women in international marriages needed a certain level of health and autonomy. The informants were all strong women with the capability of migrating to Sweden and trying to build a life here. However, dependency and stigmatization were destructive forces, whereas support, networks, and trust were capacity builders.

The dependency created by the Swedish system in an international marriage, where one of the parties is a Swedish citizen, may have severe consequences for the migrating party. These may range from social isolation to poor mental health, as well as increased vulnerability to IPV, with severe difficulties to leave the relationship. These findings are in accordance with previous research showing that without language skills and possibility to provide for themselves a person is completely dependent on the provider for the first 2 years [33]. Thus, the informants in this study had to give up their autonomy when starting a life in Sweden. This created a situation of dependency that may be difficult to end, also in a long-term perspective. Dependency implies a risk for adverse health outcomes, as autonomy is an important basic need, according to Doyal–Gough [19]. Thai women with support from their partners, who get the possibility to learn Swedish should hypothetically be able to become increasingly independent after the first bound years and to end up on one of the upper paths of our theoretical model with a good chance of being able to maintain and develop their strong selves (Figure 1). However, even for Thai women with support from their partner and network, it can be difficult to avoid moonlighting jobs or to find a job outside the service sector [34]. This also makes it difficult for them to manage on their own, if they wish to do so.

Handling prejudice turned out to be necessary for Thai women in international marriages, which agrees with previous research [5]. The informants had all been exposed to other people’s prejudice or stigmatization of them or their relationship to a Swedish man. The variety of incursions ranged from glances and frowns to being spat on or sexually harassed. Different coping strategies were developed, and the results showed that the women reacted by offering resistance, avoiding other Thai women, or by feeling ashamed of being Thai.

According to Goffman’s theory, both bodily and social collective attributes may contribute to stigmatization of Thai women [28]. Due to certain physical attributes of Thai women, it is difficult to cover one’s appearance, which led the informants to internalizing the prejudice and feeling ashamed of their origin. Such a strategy would lead to one of the lower paths of the model, with the result that it would be harder for them to maintain a strong self. The other strategy in Goffman’s theory is passing and trying to blend in, which applies to our findings of avoiding other Thai women and instead trying to reach out to other groups of people including the majority population. Such avoidance seemed to be an important strategy, since the results show that the informants had low trust in other Thai women. Offering resistance was another strategy found in our study. This required strength and support from the partner as well as from other social networks and would, according to our analysis, put the women in the strongest position with the best chances of strengthening or maintaining their strong self. However, stigma was often handled by combining different strategies and both ‘avoiding other Thai women’ and ‘internalizing shame’ can be seen as expressions of internalized social stigma.

Internalized shame and lack of social capital can be discussed in relation to social capital theory [24,26]. The results from our current and previous study show that Thai women in Sweden have access to bonding social capital within the local Thai community. However, due to internalized shame, Thai women have low trust in each other, and instead avoid other Thai women. Thus, they lose the possible benefits that bonding social capital can contribute with. Furthermore, due to their husbands’ lack of social capital and stigma they may also be excluded from networks outside their own group, i.e. bridging capital. This also means that they will have limited access to linking social capital, since this in turn depends on having access to bridging networks. This is an unfortunate situation that may leave Thai women in social isolation, sometimes with their husbands as the only social relation.

The women’s experiences and strengths were crucial for the particular path on which they ended up, after recognizing life choices. However, their partners’ attitudes and encouragement were also of great influence. Participants with support and a high level of trust in others had the best prospects for good health and autonomy. The top path in the model started with the opportunity to make an active choice to leave Thailand and move to Sweden. In Sweden, the women trusted in their own capacity and at the same time felt valued and respected in their relationships.

Our study indicated that before reaching the point of recognizing their different life choices, one step along the way was to adjust to fit in. Thai women who trusted in their own capacity often had support from their partner and access to Swedish education and a social network in Sweden. These findings are supported by our previous results showing that social capital through networks and social participation decreased poor mental health and IPV [18]. Another possibility to adjust and create a functional relationship was to evade conflicts by silence, i.e. by not discussing differences and evading confrontation to make the relationship work and decreasing the risk of being sent back to Thailand. In the third subcategory, conflict could be evaded by doing as one was told and putting one’s own needs last. These strategies resemble the strategies for maintaining harmony by women exposed to controlling or violent behavior in other settings [35]. Following this lowest path, women risk facing continued dependency of their partners and difficulties to maintain a strong self in long run.

Women who have had the adversity of ending up with a controlling or violent man could be facing a continuing dependency and be forced to stay in one of the lower paths of the model (Figure 1). However, our study also showed that some of the women actually had had the strength to leave an abusive partner after enduring the first 2 years. The Thai women in our study who came to Sweden to escape a violent relationship in Thailand were also at risk of following a path where they feared to be abandoned and thus tried to please other people including their partner, internalized the shame built into the prejudice they met, and ended up in a situation where they could not see a way out of the relationship.

Persons living in a controlling relationship have limited possibility to create social capital outside of the relationship or to get the freedom to have their own space. Thus, a controlling situation may lead to social isolation, which can be detrimental for a Thai woman without any other connections or safety nets in the country [36].

According to previous research, social isolation can be both a risk factor and a consequence of IPV [36,37]. In our survey of Thai women we found a statistically significant association between IPV and social isolation [18]. Not allowing women to work is one way for men to maintain control, as shown in a study of immigrant Vietnamese women in the US [38]. Social isolation and IPV are risk factors for poor mental health and could according to our theoretical model lead to decreased possibility for the women to maintain a strong self, leaving them with a broken self-esteem and in continued dependency [13,18].

### Methodological considerations

The current study was part of a larger project aiming to explore health and well-being of Thai women in Sweden [34]. The study was a follow-up of a survey among Thai women, with approximately 800 respondents. An open question in the questionnaire gave them the opportunity to participate in an interview study. Those who gave their name and phone number thereby gave their consent to be contacted. The use of participants from the survey can be seen as a strength since it gave access to background characteristics and other information on the informants. Most of the informants could thereby be purposively sampled to represent different socio-demographic characteristics as well as varied experience of controlling behavior and IPV. However the sampling of informants also became a challenge, since many respondents had changed phone numbers or for various reasons no longer wanted to participate in the study. The fact that the informants had stayed in Sweden for an average of 5 years, ranging from 4 to 9 years, is seen as a strength. They were all able to reflect back on a longer time period and did not have to worry about their permanent residency at the time of the interview.

In order to further enhance credibility, data collection and data analysis were continuously discussed and reflected upon in the research group. The development of focused codes, categories, subcategories, and core category was based on detailed continuous memo writing done by the first author throughout the research process. To illustrate how categories and subcategories were grounded in the data, quotes were included in the description of the results. Sampling informants living in the three largest cities in Skane county may however limit the transferability of the results to similar urban settings. The study challenges existing prejudice of Thai women in international marriages by showing the variation in experiences and illuminating the difficulties that may occur, thus supporting its originality. Resonance was enhanced by the decision to conduct an additional two interviews, after a preliminary analysis had been done. This represents an effort to ensure a more complete picture of the participants’ experiences. The study results also clearly indicated the impact of legislation on everyday life for our informants. Finally, the current findings are useful in that they indicate that support might be needed for the most isolated and abused Thai women in Sweden. Further research is needed to explore possible ways of providing such support.

### Conclusion and implications

Through the grounded theory analysis, illustrated in a theoretical model, it was possible to show the crucial role of economical, emotional and social support from partners and networks for Thai women’s possibilities for maintaining a strong self and a good health after migration. The women’s introduction to the Swedish society, their access to Swedish language education and the labor market all depend upon their partners’ goodwill, possibilities and efforts. Even if Thai women coming to Sweden have to be strong, with mental and physical resources to migrate, their situation makes them vulnerable and targets for stigmatization, exploitation, and violence. This implies a need for supporting Thai women to be more independent by giving access to language education, employment and community involvement. The current requirement for becoming a permanent resident should also be reviewed, since it may force women in international marriages to remain in less healthy relationships with controlling or abusive partners.

## Paper context

Dependency, social isolation, and stigma are risk factors for poor mental health among Thai women in international marriages. Our study shows the crucial role of economical, emotional and social support from partners/networks for Thai women to maintain a strong self and good health after migration. This implies a need for increased independency through language education, employment and community involvement, and reviewing requirements for permanent residency not to jeopardize women in international marriages’ possibilities to leave unhealthy relations.

## Appendix

**Box 1.** Example of a memo.

An example of a memo that was written in the beginning of developing the category ‘Starting in dependency’.
Thai women coming to Sweden migrate for different reasons, but all share the experience of starting their new life in dependency on someone in Sweden. For example, a man whom they are planning to marry or a relative with a job opportunity. It is often the person who made the transfer possible. Most likely a person they do not know very well. Regardless of who it is, the new situation comprises uncertainty about the future and requires that they stay with that person during the next 2 years, otherwise they risk being sent back to Thailand. How does this affect the women’s possibilities of independency and freedom further on? What options do they have if they are not treated well?

## References

[1] Wojtenko K. International marriages in Sweden: a case study of Asian women and Western men [master thesis] Lund: Lund University, Centre for east and south-east Asian studies; 2012.

[2] Tsay CL Marriage migration of women from China and Southeast Asia to Taiwan In: Jones G, Ramdas K, editors. book: (Un)tying the knot: ideal and reality in Asian marriage. Singapore: National University of Singapore; 2004.

[3] Jones G, Shen H International marriages in East and Southeast Asia: trends and research emphases. Citizens Stud. 2008;5:237–12.

[4] Chia-Wen Lu M Marriage migration in China: the enlargement of marriage markets in the era of market reforms In: Palriwala R, Uberoi P, editors. book: marriage, migration and gender. Thousand Oaks (CA): Sage Publications; 2010.

[5] Behtoui A Marriage patterns of immigrants in Sweden. J Comp Fam Stud. 2010;41:415–435.

[6] Statistics Sweden 2016 [updated 2016 2 6; cited 2016 Jan 20]. Available from: http://www.scb.se/

[7] Swedish law on foreigners (2005:716) Residence permit due to family ties, chapter 5:§3b. [In Swedish: utlänningslagen (2005:716). Uppehållstillstånd på grund av anknytning, Kapitel 5: §3b]. [cited 2016 1 31]. Available from: https://www.lagboken.se/dokument/lagar-och-forordningar/879/utlanningslag-2005_716?pageid=64244&search=uppeh%C3%A5llstillst%C3%A5nd%20anknytning%20tredjelandsmedborgare

[8] Leong F Encyclopaedia of counselling. Thousand Oaks (CA): Sage Publications; 2008.

[9] Root M Love’s revolution: interracial marriage. Philadelphia (PA): Temple University Press; 2001.

[10] Jedlicka D Globalization of romance: love and marriage in the global village. Fam Sci Rev. 2000;13:28–38.

[11] Constable N Romance on a global stage: pen pals, virtual ethnography, and “mail order” marriages. Berkeley: University of California; 2003.

[12] Simons L Marriage, migration and markets: international matchmaking and international feminism. Denver: University of Denver; 2001.

[13] Bhugra D Migration and mental health. Acta Psychiatr Scand. 2004;109:243–258. 15008797 10.1046/j.0001-690x.2003.00246.x

[14] Cantor-Graae E, Selten JP Schizophrenia and migration: a meta-analysis and review. Am J Psychiatry. 2005;162:12–24. 15625195 10.1176/appi.ajp.162.1.12

[15] van Bergen DD, van Balkom AJLM, Smit JH, et al “I felt so hurt and lonely”: suicidal behavior in South Asian-Surinamese, Turkish, and Moroccan women in the Netherlands. Transcult Psychiatry. 2012;49:69–86. 22194345 10.1177/1363461511427353

[16] Liamputtong P, Naksook C Life as mothers in a new land: the experience of motherhood among Thai women in Australia. Health Care Women Int. 2003;24:650–668. 14627211 10.1080/07399330390217725

[17] Jin X, Keat JE The effects of change in spousal power on intimate partner violence among Chinese immigrants. J Interpers Violence. 2009;10:1–16. 10.1177/0886260509334283 19423746

[18] Fernbrant C, Emmelin M, Essén B, et al Intimate partner violence and poor mental health among Thai women residing in Sweden. Glob Health Action. 2014;7:24991. 10.3402/gha.v7.24991 PMC4166544 25231099

[19] Doyal L, Gough I A theory of human need. Basingstoke: Macmillan; 1991.

[20] Sen A Resources, values and development. Oxford: Blackwell; 1984.

[21] Nussbaum MC Nature, function and capability: aristotle on political distribution. Oxford Studies in Ancient Philosophy. Oxford: JSTOR; 1988 p. 145–184. Supplementary Volume 1.

[22] Gough I Lists and thresholds: comparing our theory of human need with Nussbaum’s capabilities approach. LSE Research Online; 2003 Available from: http://eprints.lse.ac.uk

[23] Putnam RD Bowling alone: the collapse of revival of American community. New York: Simon & Schuster; 2000.

[24] Portes A Downsides of social capital. Proc Natl Acad Sci. 2014;111:18407–18408. 25535346 10.1073/pnas.1421888112 PMC4284550

[25] Eriksson M Social capital health and community action – implications for health promotion. Umeå: Umeå University; 2010.

[26] Szreter S, Woolcock M Health by association? Social capital, social theory, and the political economy of public health. Int J Epidemiol. 2004;33:650–667. 15282219 10.1093/ije/dyh013

[27] Scrambler G Sociological theory and medical sociology. London and New York: Tavistock Publications; 1987 p. 135–136, 142–143.

[28] Goffman E Stigma: notes on the management if spoiled identity. Harmondsworth: Penguin; 1968.

[29] Charmaz K Constructing grounded theory. 2nd ed. London: Sage Publications; 2014.

[30] Blumer H What is wrong with social theory? Am Sociol Rev. 1954;18:3–10.

[31] Clarke AE Situational analysis. Grounded theory after the postmodern turn. California: Sage Publications; 2005.

[32] Dahlgren L, Emmelin M, Winkvist A Qualitative methodology for international public health. 2nd ed. Umeå: Epidemiology and Public Health Sciences, Department of Public Health and Clinical Medicine, Umeå University; 2007.

[33] Haandrikman K Binational marriages in Sweden: is there an EU effect? Popul Space Place. 2014;20:177–199.

[34] Stafström et al HELMI – Health, Migration and Integration. Somali and Thai women’s health and labor market connection in Sweden. Somaliska och thailändska kvinnors hälsa och arbetsmarknadsanknytning [Report by Malmö University, Lund University and Uppsala University]. Malmö: Malmö University; 2012.

[35] Hayati EN, Eriksson M, Hakimi M, et al ‘Elastic band strategy’: women’s lived experience of coping with domestic violence in rural Indonesia. Glob Health Action. 2013;6:1–12. Available from. doi:10.3402/gha.v6i0.18894 PMC3536939 23336615

[36] Raj A, Silverman JG Immigrant south Asian women at greater risk for injury from intimate partner violence. Am J Public Health. 2003;93:435–437. 12604489 10.2105/ajph.93.3.435 PMC1447758

[37] James SE, Johnson J, Raghavan C “I couldn’t go anywhere”: contextualizing violence and drug abuse: a social network study. Violence Against Women. 2004;10:991–1014.

[38] Morash M, Bui H, Zhang Y, et al Risk factors for abusive relationships. A study of Vietnamese American immigrant women. Violence Against Women. 2007;13:653–675. 17600304 10.1177/1077801207302044

